# The effect of age and mitigation strategies during hot water immersion on orthostatic intolerance and thermal stress

**DOI:** 10.1113/EP090993

**Published:** 2023-02-27

**Authors:** Charles J. Steward, Campbell Menzies, Neil D. Clarke, Amy E. Harwood, Mathew Hill, Christopher J. A. Pugh, C. Douglas Thake, Tom Cullen

**Affiliations:** ^1^ Centre for Sport Exercise and Life Sciences Coventry University Coventry UK; ^2^ Cardiff School of Sport & Health Sciences Cardiff Metropolitan University Cardiff UK

**Keywords:** heat, orthostasis, safety

## Abstract

Hot water immersion improves cardiovascular health and sporting performance, yet its adverse responses are understudied. Thirteen young and 17 middle‐aged adults (*n* = 30) were exposed to 2 × 30 min bouts of whole‐body 39°C water immersion. Young adults also completed cooling mitigation strategies in a randomized cross‐over design. Orthostatic intolerance and selected physiological, perceptual, postural and cognitive responses were assessed. Orthostatic hypotension occurred in 94% of middle‐aged adults and 77% of young adults. Young adults exhibited greater dizziness upon standing (young subjects, 3 out of 10 arbitrary units (AU) vs. middle‐aged subjects, 2 out of 10 AU), with four terminating the protocol early owing to dizziness or discomfort. Despite middle‐aged adults being largely asymptomatic, both age groups had transient impairments in postural sway after immersion (*P* < 0.05), but no change in cognitive function (*P* = 0.58). Middle‐aged adults reported lower thermal sensation, higher thermal comfort, and higher basic affect than young adults (all *P* < 0.01). Cooling mitigation trials had 100% completion rates, with improvements in sit‐to‐stand dizziness (*P* < 0.01, arms in, 3 out of 10 AU vs. arms out, 2 out of 10 AU vs. fan, 4 out 10 AU), lower thermal sensation (*P* = 0.04), higher thermal comfort (*P* < 0.01) and higher basic affect (*P* = 0.02). Middle‐aged adults were predominantly asymptomatic, and cooling strategies prevented severe dizziness and thermal intolerance in younger adults.

## Abstract (highlights)

- **What is the central question of this study?**
  The aim was to characterize adverse responses to whole‐body hot water immersion and to investigate practical strategies to mitigate these effects.
- **What is the main finding and its importance?**
  Whole‐body hot water immersion induced transient orthostatic hypotension and impaired postural control, which recovered to baseline within 10 min. Hot water immersion was well tolerated by middle‐aged adults, but younger adults suffered from a greater frequency and severity of dizziness. Cooling the face with a fan or not immersing the arms can mitigate some of these adverse responses in younger adults.

## 1 INTRODUCTION

Hot water immersion has the potential to improve cardiovascular health (Brunt et al., 2016; Ukai et al., 2020) and sporting performance (Philp et al., 2022; Zurawlew et al., 2016), sharing numerous benefits with exercise training (Cullen et al., 2020). However, several studies suggest that this method of heating can be uncomfortable and difficult to tolerate (Hoekstra et al., 2018; Zurawlew et al., 2016). Furthermore, prolonged hot water immersion can result in negative symptoms, including dizziness, syncope, nausea, headaches and vomiting (Horvath & Botelho, 1949; James et al., 2021; Menzies et al., 2022). In some circumstances, the deleterious effects of heating can also contribute to transient declines in postural control (Mtibaa et al., 2018) and cognitive function (Malcolm et al., 2018; Sun et al., 2012), with previous research linking these factors to the risk of falls in older adults (Johansson et al., 2017). Although some of these adverse symptoms have been recognized for a long time (Horvath & Botelho, 1949), many of the more recent studies investigating hot water immersion appear to overlook these important safety considerations. Indeed, adverse events and safety concerns often receive only a single passing comment in many papers (Hooper, 1999; James et al., 2021; Zurawlew et al., 2016).

Public guidance for the safe use of hot water immersion includes limits on temperature and duration to 40°C and 15 min, respectively (e.g., National Spa & Pool Institute, 1988; The Pool & Hot Tub Alliance, 2020). Yet, an increasing number of studies have demonstrated improvements in health using hot water immersion protocols that drastically exceed these limits, with durations ranging from 20 to 120 min and temperatures from 39 to 48°C (e.g., Brunt et al., 2016; Kojima et al., 2018; Rodrigues et al., 2020; Ruddock et al., 2016). In some studies, it appears that these protocols were achievable only by using precautionary methods that might not be realistic or usable outside the laboratory, including monitoring for high rectal temperatures (Brunt et al., 2016) and assistance by researchers to mitigate dizziness and prevent syncope (Hooper, 1999). The risk of heat‐induced orthostatic intolerance appears to be the most important issue from a safety perspective, but the majority of studies investigating this phenomenon have used a tilt table (Horvath & Botelho, 1949; Lind et al., 1968; Minson et al., 1999). It is unclear how these responses would translate into individuals exiting a bath or hot tub, moving from a sitting to standing posture in free‐living conditions. As such, we consider it important to assess the safety and tolerability of hot water immersion thoroughly, in an applied manner, with a higher level of ecological validity.

It is well established that thermal intolerance (Kenney & Hodgson, 1987) and orthostatic intolerance (Goswami et al., 2017) are altered by age. In response to an orthostatic challenge following passive heating, older adults have been shown to have a greater reduction in cerebral blood flow and slower corrections in blood pressure when compared with younger adults (Lucas et al., 2008). Accordingly, characterization of the safety and tolerability of hot water immersion protocols should be done separately for different age groups.

Given that hot water immersion can lead to adverse responses and be difficult to tolerate for some populations, it is important to investigate practical strategies to mitigate these risks. Within healthy young male adults, Mansfield et al. (2021) recently showed that fan cooling during hot water immersion could improve thermal comfort without reducing some of the beneficial effects, such as the acute anti‐inflammatory response. Skin cooling could be a possible countermeasure to combat impaired orthostatic control in the heat attributable to reductions in venous blood pooling and increased peripheral vascular resistance (Durand et al., 2004; Wilson et al., 2002). Likewise, different heating stimuli during hot water immersion can be achieved through manipulation of the water depth, which is varied within the literature [e.g., chest deep (Galbreath et al., 1999) vs. neck deep (Horvath & Botelho, 1949)], and potentially impacts the risk of orthostasis and heat intolerance. However, the effects of practical local skin‐cooling strategies on orthostatic intolerance and other adverse events following hot water immersion are yet to be investigated.

The aims of this study were as follows: (1) to investigate the effect of whole‐body hot water immersion on orthostatic intolerance and selected physiological, perceptual, postural and cognitive responses in healthy young and middle‐aged adults; and (2) to assess whether two practical mitigation strategies of facial fan cooling and non‐submersion of the arms mitigated any adverse responses in younger adults.

## 2 METHODS

### 2.1 Participants

A convenience sample of 13 young adults (seven male and six female; mean ± SD: age, 25 ± 3 years; body mass, 71.9 ± 15.1 kg; height, 1.72 ± 0.10 m; and body mass index 24.2 ± 3.7 kg/m^2^) and 17 middle‐aged adults (10 male and seven female; mean ± SD: age, 54 ± 6 years; body mass, 82.2 ± 15.5 kg; height, 1.69 ± 0.08 m; and body mass index 28.7 ± 3.9 kg/m^2^) participated in the present study. All participants were non‐smokers, had no history of syncope and were not taking any medication. Female participants in the younger cohort were tested in the early follicular phase of the menstrual cycle (days 1–7) to control for effects of the menstrual cycle on core temperature and blood pressure regulation (Charkoudian et al., 2017), and women in the middle‐aged cohort were post‐menopausal. Participants were instructed to refrain from strenuous exercise for 24 h and caffeine intake for 6 h before the experimental trials and to replicate their diet on the day of testing for each visit. Before data collection, participants were briefed, and they provided written informed consent for the study, which received ethical approval from Coventry University (P109747) and conformed to the standards of the *Declaration of Helsinki*, with the exception of prior registration in a public database.

### 2.2 Experimental design

**FIGURE 1:**
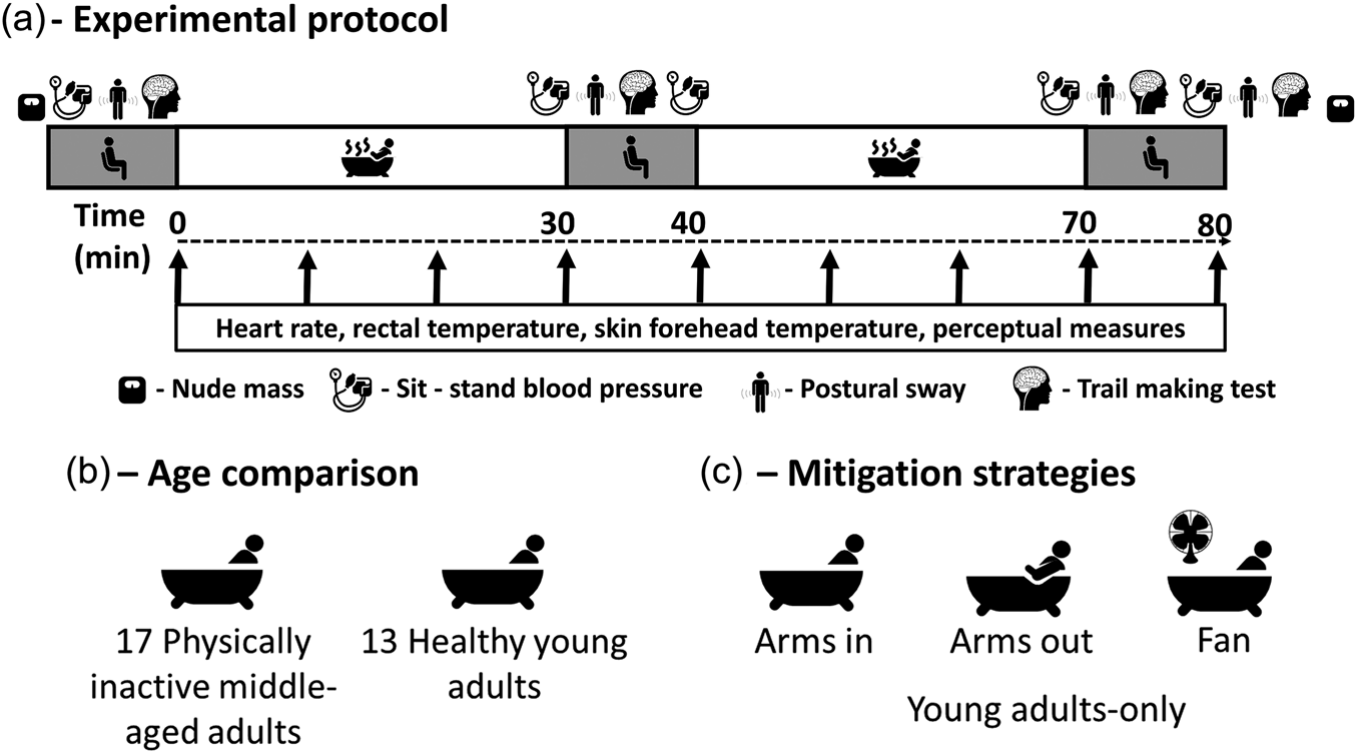
(a) Experimental protocol representing the time course of immersion and the measurements taken. (b) Age comparison: Arms in only, young adults versus middle‐aged adults. (c) Mitigation strategies: young adults only, with two mitigation strategies: Arms out versus Arms in immersion; and Fan, versus Arms in immersion.

The study design consisted of a single whole‐body hot water immersion visit for both young and middle‐aged adults to assess the effect of age. Young adults then completed two further visits to assess the effect of specific mitigation strategies. These three test sessions completed by the young adults were completed in a randomized cross‐over design. Allocation of the order of test sessions was conducted using a randomization sequence in Excel. All sessions were conducted at the same time of day (±2 h), with a minimum washout period of 72 h between visits. After familiarization with all procedures, participants were immersed in 39.0 ± 0.1°C water for 60 min, split into two bouts of 30 min separated by 10 min of rest in ambient conditions (∼20°C) (Figure 1a). This temperature and duration were chosen in line with hot water immersion protocols within the literature (e.g., Leicht et al., 2019) and following high rates of intolerance after 30 min of 40°C immersion with the arms submerged observed during pilot testing. The 10 min rest in ambient conditions was included to allow the early identification of sit‐to‐stand (Sit‐Stand) presyncope and to assess the effects of immersion duration on orthostatic intolerance, without the need for multiple visits. All participants were immersed up to the armpit with arms submerged (Arms in) for the age comparison (Figure 1b), and the young participants also completed two mitigation conditions involving immersion up to the armpit with facial fan cooling at a wind speed of ∼1.5 m/s (Fan) and immersion to the armpit without submersion of the arms (Arms out) for the condition comparison (Figure 1c). The condition comparison was performed only within the younger participants, because previous studies have reported symptomatic responses in younger but not older adults (Vaddadi et al., 2007), which we also observed through our own pilot testing.

### 2.3 Experimental protocol and procedures

Upon arrival at the laboratory, participants consumed 5 mL/kg of water room temperature within 5 min to standardize hydration before commencing the experimental protocol (Figure 1a).

#### 2.3.1 Orthostatic hypotension

Orthostatic hypotension was measured and defined according to clinical guidelines (Freeman et al., 2011) as a reduction of ≥20 mmHg in systolic blood pressure (SBP) or ≥10 mmHg in diastolic blood pressure (DBP) when compared with values obtained at baseline, before the immersion period. Seated blood pressure was recorded using an automated sphygmomanometer (M3 blood pressure monitor; Omron, Kyoto, Japan) whilst seated on a chair or during the final 5 min of immersion. Baseline blood pressure was recorded in triplicate at the start of the protocol following a 5 min period of quiet rest. Participants were instructed to stand up gradually upon the sensation of cuff inflation, with a single measure of standing blood pressure taken. Upon standing, participants reported symptoms of dizziness on the same 0–10 scale used for indices of heat illness (Coris et al., 2006), with standing dizziness being recorded as the maximum dizziness reported. Owing to the transient nature of standing dizziness, participants reporting scores ranging from 7 to 10 were given the option to continue if their symptoms had subsided by the time they had to re‐enter the water.

#### 2.3.2 Thermophysiological measures

Nude body mass was recorded using electronic weighing scales (Seca; Bodycare, UK) before and after the experimental protocol to estimate the whole‐body sweat rate. Rectal temperature was monitored at 10 min intervals, using a rectal thermometer (Grant Instruments, UK). Simultaneous measurements of heart rate (Polar FT1; Polar, Kempele, Finland) and skin forehead temperature (skin thermistor; Grant Instruments, UK) were also taken at 10 min intervals.

#### 2.3.3 Perceptual measures

Thermal comfort, thermal sensation and basic affect were measured every 10 min on scales ranging from +5 (very comfortable, hot and very good, respectively) to −5 (very uncomfortable, cold and very bad, respectively) modified from Epstein and Moran (2006) and Williams et al. (2008) for ease of participant understanding. Symptoms of dizziness, confusion, tiredness, nausea and headaches were monitored on a 0–10 scale, with 0 representing no symptoms, 3 representing mild symptoms, 5 representing moderate symptoms, 7 representing severe symptoms and 10 representing have to stop (Coris et al., 2006).

#### 2.3.4 Postural sway

Centre‐of‐pressure measures of postural sway assessed during quiet standing were used to characterize postural control. This was included because poorer postural control has been observed in individuals with orthostatic intolerance (Claydon & Hainsworth, 2005). Participants stood barefoot, with their feet together and hands clasped in front of the body, on a force platform (AMTI, Watertown, MA, USA) and were instructed to stand quietly for 30 s while looking ahead at a black circle at eye level on a wall, 1.5 m away. Baseline results were averaged over three trials, with participants stepping off the plate for 30 s between each trial. Data were sampled at 100 Hz, with outcome measures including the total path length (in centimentres) of the centre of pressure and its maximal displacement in the anteroposterior and mediolateral directions.

#### 2.3.5 Trail‐making test

The psychology experiment building language (PEBL) trail‐making test (Mueller & Piper, 2014) was used as a measure of executive function (Sánchez‐Cubillo et al., 2009). This test consists of part A and B, where participants first click numbers in ascending order from 1 to 26 followed by a second task where numbers and letters are clicked in alternation (1‐A‐2‐B etc.) up to 13‐M. The trail‐making test is a valid measure of executive function (Sánchez‐Cubillo et al., 2009) and has high levels of task duration retest reliability for part A of 0.76–0.89 and part B 0.86–0.94 (Wagner et al., 2011). Participants were instructed to complete the trial as quickly and accurately as possible. The main outcome measure from this test was the total duration to complete parts A and B. The secondary outcome was task accuracy, defined as the minimum number of clicks to complete each trial divided by the number of clicks made.

### 2.4 Statistical analysis

Data are reported as means and SDs for physiological data and as medians with interquartile ranges for perceptual data, unless stated otherwise. Statistical significance was accepted as α < 0.05. Analysis was conducted in RStudio using the functions and packages stated below, with an example of the code found in the Supporting Information. Physiological data were examined using linear mixed‐effects modelling using the *lmer* function from the *lme4* package, with fixed effects including condition, time and their interaction and with random effects accounting for each individual whilst controlling for condition and time effects. Although this study was not designed to assess sex differences, sex was added as a fixed effect for supplementary analysis. These results are available in the Supporting Information and are included in the present manuscript only where significant sex × time × group interactions were observed. The significance of fixed effects was determined using the *anova* function from the *stats* package, according to the approximations by Kenward and Roger (1997) for the denominator degrees of freedom. Conditional *R* ^2^ (*R* ^2^_c_) were calculated using the *r.squaredGLMM* function from the *MuMIn* package and provided the variance explained by fixed effects within the model, in line with Nakagawa and Schielzeth (2013). Additionally, effect sizes (*d*) were calculated for the main effects of condition (shown relative to the Arms in condition for Fan and Arms out, separately) and group, by dividing the mean differences by the square root of the sum of random variance components as suggested by Westfall et al. (2014). Data were not remodelled to other distributions, despite some violations of normality in the residuals, owing to the robustness of linear models to this violation (Knief & Forstmeier, 2020; Schielzeth et al., 2020).

Perceptual data were examined using cumulative link mixed models using the *clmm* function from the *ordinal* package, fitting fixed and random effects in the same manner as the physiological data. Where the model failed to converge in the condition comparison, random effects were simplified by the removal of group–participant and time–participant interactions. The *Anova.clmm* function from the *RVAideMemoire* package was used to perform Wald type II χ^2^ tests to evaluate the significance of the fixed effects. As a measure of variance explained by the model, Nagelkerke pseudo‐*R* ^2^ (*R* ^2^_n_) was calculated using the *nagelkerke* function within the *rcompanion* package by comparing the fitted model with a null model with only random effects. The proportional odds assumption was checked using the graphical method described by Harrell (2001).

Post hoc testing of pairwise comparisons of estimated marginal means with Bonferroni adjustment was conducted using the *emmeans* function from the *emmeans* package to evaluate differences between group/condition where significant interaction effects were detected.

To investigate potential associations between variables, exploratory analysis was conducted by performing correlations at the 30 min time point to allow for the inclusion of participants who could not complete the protocol, with removal of data at 70 min to maintain independent observations within the data. Correlations were performed using the *rcorr* function from the *Hmisc* package, with Spearman's correlations calculated for associations involving thermal sensation, thermal comfort, affect and standing dizziness, and with Pearson's correlations calculated for all other relationships. All raw data and the code used to analyse them are provided as the Supporting Information.

## 3 RESULTS

### 3.1 Completion and orthostatic hypotension

The proportion of participants who successfully completed the protocol along with the proportion who reached the clinical threshold for orthostatic hypotension immediately after immersion is shown in Table 1. Four young participants (4 of 13; 31%) were unable to complete the Arms in condition owing to severe dizziness upon standing after 30 min (two males) or intolerance during the second heating bout (two females; 60 and 65 min). Moreover, despite no participants demonstrating orthostatic hypotension at rest upon enrolment in the study, almost all participants (87%) reached the threshold for orthostatic hypotension during the protocol at either 30 or 70 min or both, following immersion in the Arms in condition.

**TABLE 1:** Summary data for completion, orthostatic hypotension, standing dizziness and symptoms of heat illness responses to the hot water immersion protocol.

Parameter	Middle‐aged, Arms in	Young, Arms in	Young, Arms out	Young, Fan
Completion	100% (17/17)	69% (9/13)	100% (13/13)	100% (13/13)
Orthostatic hypotension	94% (16/17)	77% (10/13)	62% (8/13)	46% (6/13)
Standing dizziness^1^, ^2^, ^4^, ^5^				
30 min (a.u.)	0 (0, 3)	3 (1, 6)	1 (0, 3)	3 (1, 5)
70 min (a.u.)	2 (0, 5)	2 (2, 4)	3 (1, 4)	4 (1, 6)
Symptoms of heat illness				
Confusion	14/1/1/1/0	12/0/1/0/0	13/0/0/0/0	11/1/1/0/0
Dizziness	13/2/1/1/0	9/2/1/1/0	13/0/0/0/0	10/2/1/0/0
Headaches	12/5/0/0/0	11/1/1/0/0	10/2/1/0/0	9/3/0/1/0
Nausea	15/1/1/0/0	10/2/0/1/0	13/0/0/0/0	10/3/0/0/0
Tiredness	12/2/2/1/0	8/2/2/1/0	8/3/2/0/0	8/3/1/1/0
Total	66/11/5/3/0	50/7/5/3/0	57/5/3/0/0	48/12/3/2/0

*Note*: Standing dizziness scores are displayed as the median (lower quartile, upper quartile). Frequencies of symptoms of heat illness in each condition are summarized as the peak response across the protocol across all participants for scores of 0/1–2/3–4/5–6/7–10 (*n* = 13 young, *n* = 17 middle‐aged). Significant main effects are denoted as follows:

^1^ time (age comparison)

^2^ group (age comparison)

^4^ time (condition comparison)

^5^ = condition (condition comparison).

**FIGURE 2:**
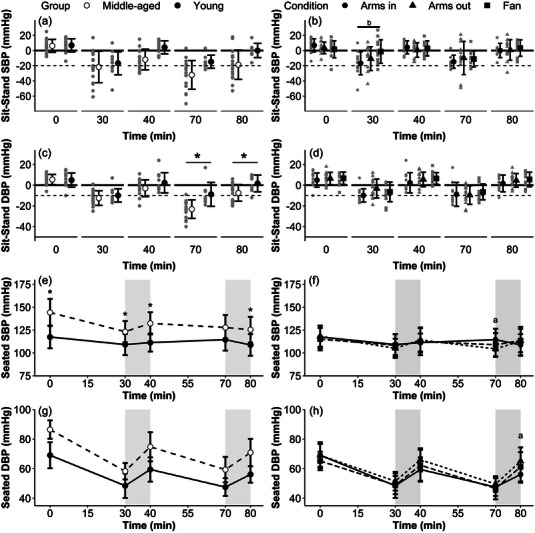
Blood pressure responses over time for age comparisons of change from seated rest in SBP upon standing (a), change from seated rest in DBP upon standing (c), seated SBP (e) and seated DBP (g); and for condition comparisons of change from seated rest in SBP upon standing (b), change from seated rest in DBP upon standing (d), seated SBP (f) seated DBP (h). Significance is denoted as follows: *young versus middle‐aged; ^a^Arms in versus Arms out; and ^b^Arms in versus Fan. Dashed horizontal lines (in a–d) represent the clinical thresholds for orthostatic hypotension (*n* = 13 young, *n* = 17 middle‐aged). Shaded grey areas represent the non‐immersion periods during the protocol. Filled circles display the same data from younger participants in the Arms in condition for both the age comparisons (left‐hand panels) and condition comparisons (right‐hand panels). Abbreviations: DBP, diastolic blood pressure; SBP, systolic blood pressure.

In the age comparison for Sit‐Stand blood pressure (BP), the change in SBP from seated rest was greatest immediately after immersion (30 and 70 min), with larger responses observed in the middle‐aged adults resulting in significant main effects of time (*P* < 0.001) and group (*P* = 0.02, *d* = 0.81) but not their interaction (*P* = 0.05, *R* ^2^_c_ = 0.43; Figure 2a). Sit‐Stand DBP displayed a similar pattern, with greater reductions in DBP in the middle‐aged participants after the second bout of immersion (70 and 80 min; Figure 2c), with significant main effects of time (*P* < 0.001), group (*P* = 0.01, *d* = 0.80) and their interaction (*P* < 0.001, *R* ^2^_c_ = 0.56). In the condition comparison, the same reductions in Sit‐Stand BP were observed after immersion for both SBP and DBP, resulting in a significant main effect of time for both variables (*P* < 0.001; Figure 2b,d). However, there was no effect of condition for either SBP (*P* = 0.45, Arms out *d* = 0.04, Fan *d* = 0.22) or DBP (*P* = 0.27, Arms out *d* = 0.33, Fan *d* = 0.38), despite an interaction effect showing a difference in Sit‐Stand SBP (*P* = 0.004, *R* ^2^_c_ = 0.27) between the Arms in and Fan conditions after 30 min. Sit‐Stand BP also showed significant sex × time × group interactions for both SBP (*P* = 0.02, *R* ^2^_c_ = 0.50) and DBP (*P* = 0.04, *R* ^2^_c_ = 0.59) within the age comparison, with greater reductions in both SBP and DBP for women in the middle‐aged group and for men in the younger group (disaggregated mean responses over time by sex can be found in the Supporting Information).

Seated SBP and DBP reduced in both young and middle‐aged groups after immersion, resulting in significant main effects of time, group and the time × group interaction for seated SBP (all *P* < 0.01, *d* = 1.44, *R* ^2^_c_ = 0.42; Figure 2e) and significant main effects for time and group (both *P* < 0.001, *d* = 1.64), but not their interaction (*P* = 0.08, *R* ^2^_c_ = 0.66), for seated DBP (Figure 2g) in the age comparison. For the condition comparison, there was no effect of condition for seated SBP (*P* = 0.67, Arms out *d* = 0.11, Fan *d* = 0.06; Figure 2f); however, significant main effects of time (*P* < 0.001), condition (*P* = 0.02, Arms out *d* = 0.44, Fan *d* = 0.03) and the time × condition interaction (*P* = 0.02, *R* ^2^_c_ = 0.49) were observed for seated DBP with Arms in, resulting in the greatest decreases in DBP (Figure 2h).

Standing dizziness scores after immersion are summarized in Table 1, with higher dizziness scores in the younger participants compared with the middle‐aged participants after the first bout of immersion and an increase in dizziness after the second immersion bout. Non‐immersion time points (times 0, 40 and 80 min), which all had dizziness scores of 0 (0, 0) a.u., showed the transient nature of dizziness symptoms. Owing to a lack of model convergence, these time points were dropped from the statistical analysis, which showed a significant main effect of time (*P* < 0.001) and group (*P* = 0.02) but no interaction (*P* = 0.64, *R* ^2^_n_ = 0.94) for the age comparison and a significant main effect of time (*P* = 0.003) and condition (*P* < 0.001) but no interaction (*P* = 0.91, *R* ^2^_n_ = 0.25) for the condition comparison.

### 3.2 Thermophysiological responses

**FIGURE 3:**
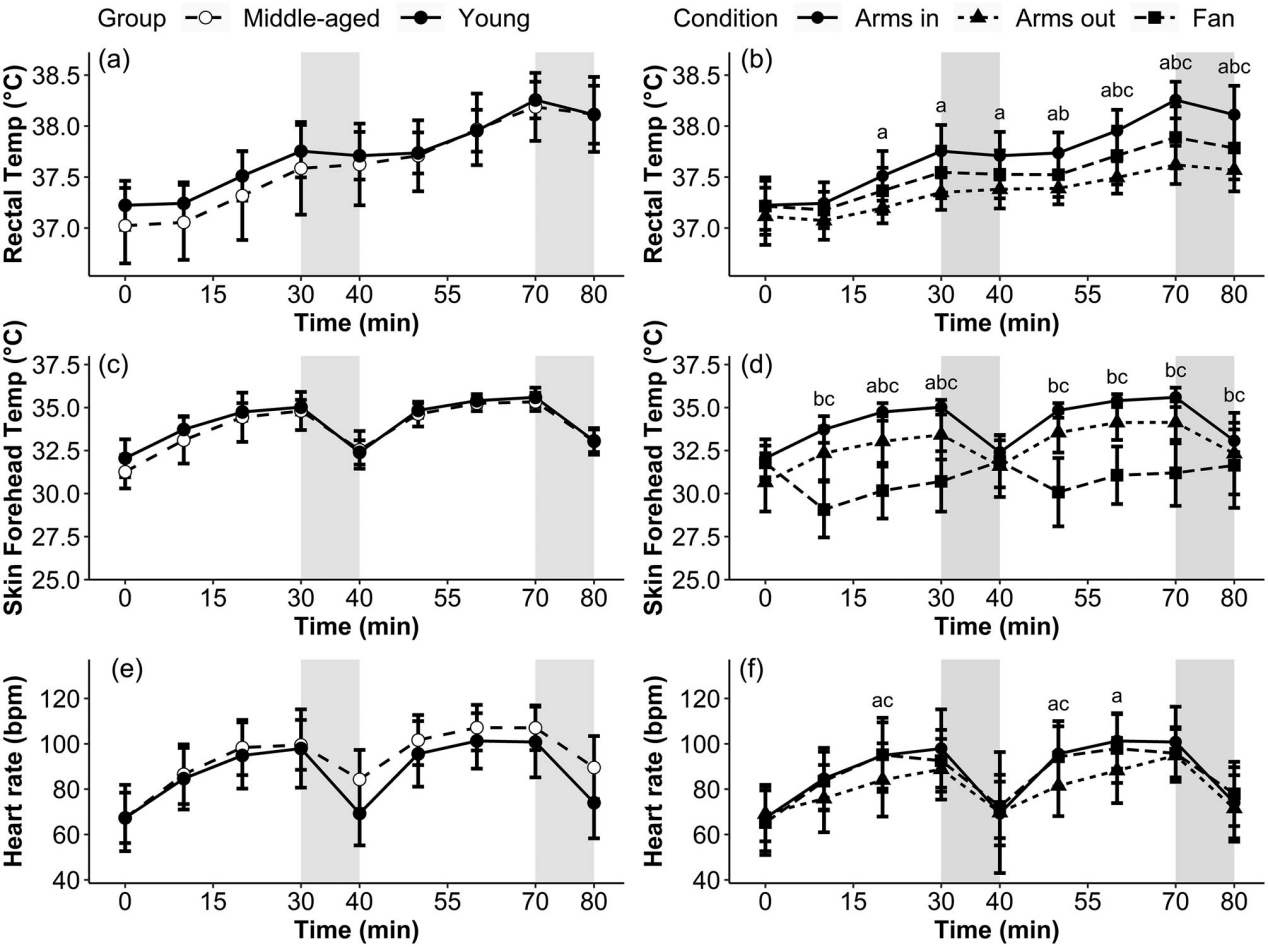
Thermophysiological responses over time for the age comparisons of rectal temperature (a), skin forehead temperature (c) and heart rate (e); and condition comparisons of rectal temperature (b), skin forehead temperature (d) and heart rate (f). Significance is denoted as follows: *young versus middle‐aged; ^a^Arms in versus Arms out; ^b^Arms in versus Fan; and ^c^Arms out versus Fan (*n* = 13 young, *n* = 17 middle‐aged). Shaded grey areas represent the non‐immersion periods during the protocol. Filled circles display the same data from younger participants in the Arms in condition for both the age (left‐hand panels) and condition (right‐hand panels).

Rectal temperature, skin forehead temperature and heart rate are presented in Figure 3. The protocol increased rectal temperature to 38.3 ± 0.2 and 38.2 ± 0.3°C at 70 min in the Arms in condition for the younger and middle‐aged participants, respectively, resulting in a significant main effect of time (*P* < 0.001) but not group (*P* = 0.34, *d* = 0.19; Figure 3a). Meanwhile, both the Arms out (70 min, 37.6 ± 0.2°C) and Fan (70 min, 37.9 ± 0.3°C) conditions increased rectal temperature to a lesser extent, with significant main effects for time, condition and their interaction (all *P* < 0.001, Arms out *d* = 1.44, Fan *d* = 0.68, *R* ^2^_c_ = 0.60) observed for the condition comparison (Figure 3b). Skin forehead temperature increased over time (*P* < 0.001) but did not differ between age groups (*P* = 0.18, *d* = 0.25; Figure 3b), and heart rate also increased throughout the protocol, displaying significant effects of time and a group × time interaction (both *P* < 0.01, *R* ^2^_c_ = 0.48), but no effect of group (*P* = 0.30, *d* = 0.47; Figure 3c). The Fan condition resulted in an initial decrease in skin forehead temperature, with smaller increases observed in the Arms out condition compared with Arms in, resulting in a significant main effect of condition, time and their interaction (all *P* < 0.001, Arms out *d* = 0.85, Fan *d* = 2.20, *R* ^2^_c_ = 0.59; Figure 3d). Likewise, heart rate showed significant main effects for condition, time and their interaction (all *P* < 0.001, Arms out *d* = 0.45, Fan *d* = 0.07, *R* ^2^_c_ = 0.37), with the smallest increases observed in the Arms out condition (Figure 3f). The decrease in nude mass from baseline was similar in all conditions (middle‐aged: −0.6 ± 0.3 kg; young: Arms in, −0.7 ± 0.5 kg; Arms out, −0.5 ± 0.2 kg; Fan, −0.7 ± 0.2 kg), resulting in a significant main effect of time for both the age and condition comparisons (*P* < 0.001), but no group effect (*P* = 0.07, *d* = 0.82) or condition effects (*P* = 0.88, Arms out *d* = 0.01, Fan *d* < 0.01).

### 3.3 Perceptual responses

**FIGURE 4:**
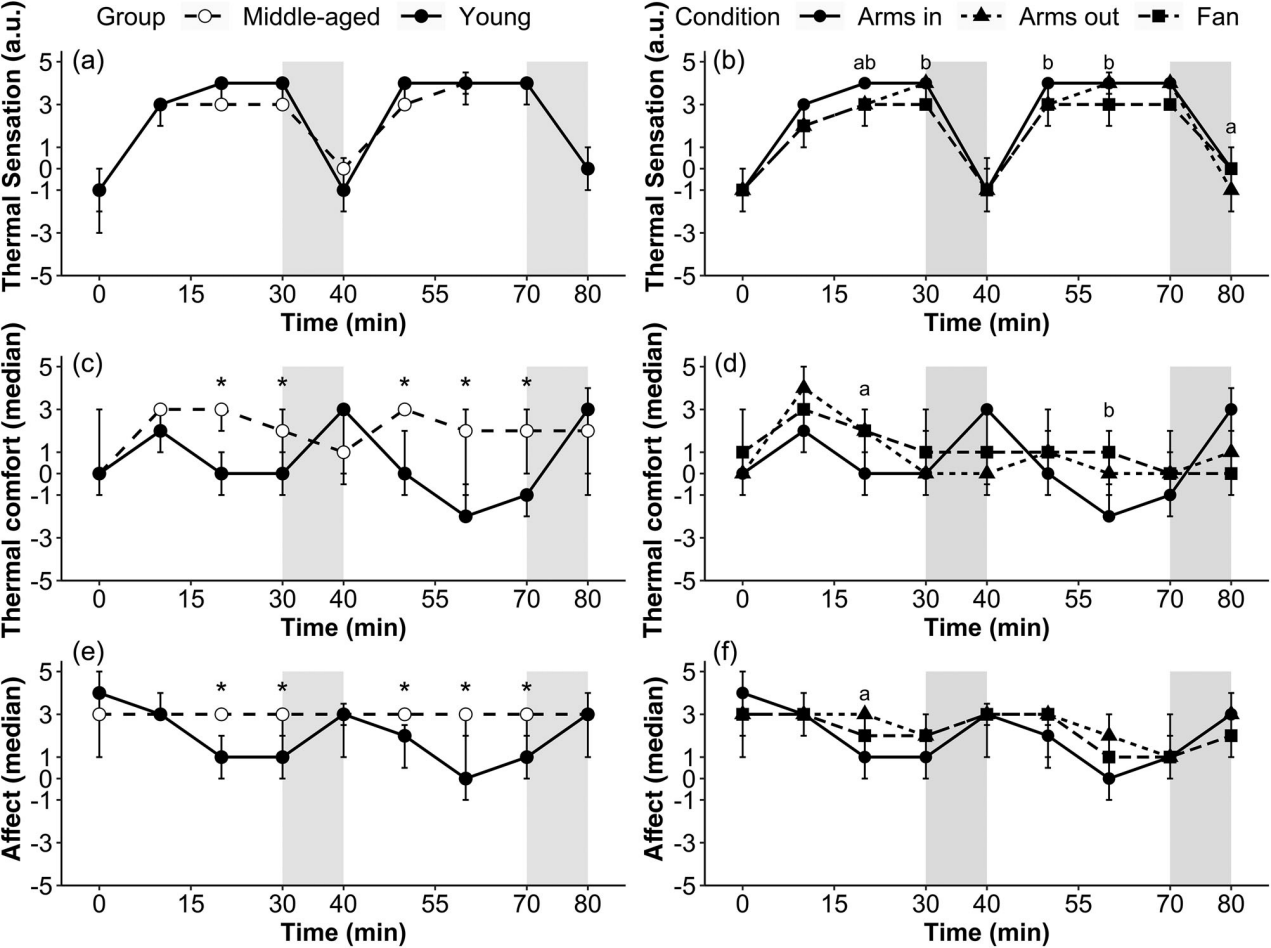
Perceptual responses over time for the age comparison of thermal sensation (a), thermal comfort (c) and affect (e); and condition comparison of thermal sensation (b), thermal comfort (d) and affect (f). Significance is denoted as follows: *young versus middle‐aged; ^a^Arms in versus Arms out; and ^b^Arms in versus Fan; (*n* = 13 young, *n* = 17 middle‐aged). Shaded grey areas represent the non‐immersion periods during the protocol. Filled circles display the same data from younger participants in the Arms in condition for both the age (left‐hand panels) and condition (right‐hand panels).

Summary data for thermal sensation, thermal comfort and affect are displayed in Figure 4, with all variables displaying main effects of time, group and their interaction (all *P* < 0.05; thermal sensation, *R* ^2^_n_ = 0.79; thermal comfort, *R* ^2^_n_ = 0.33; affect, *R* ^2^_n_ = 0.32) for the age comparison and for time, condition and their interaction (all *P* < 0.05; thermal sensation, *R* ^2^_n_ = 0.81; thermal comfort, *R* ^2^_n_ = 0.32; affect, *R* ^2^_n_ = 0.33) for the condition comparison. Middle‐aged participants reported lower values of thermal sensation, accompanied by higher thermal comfort and affect scores than younger participants. In the condition comparison, Arms in resulted in increased thermal sensation with lower thermal comfort and affect when compared with both the Arms out and Fan conditions. Thermal sensation showed a sex × time × condition interaction in the condition comparison (*P* = 0.03, *R* ^2^_n_ = 0.22), with men generally feeling hotter in both the Arms in and Arms out conditions (disaggregated median responses over time by sex can be found in the Supporting Information).

### 3.4 Postural sway

**FIGURE 5:**
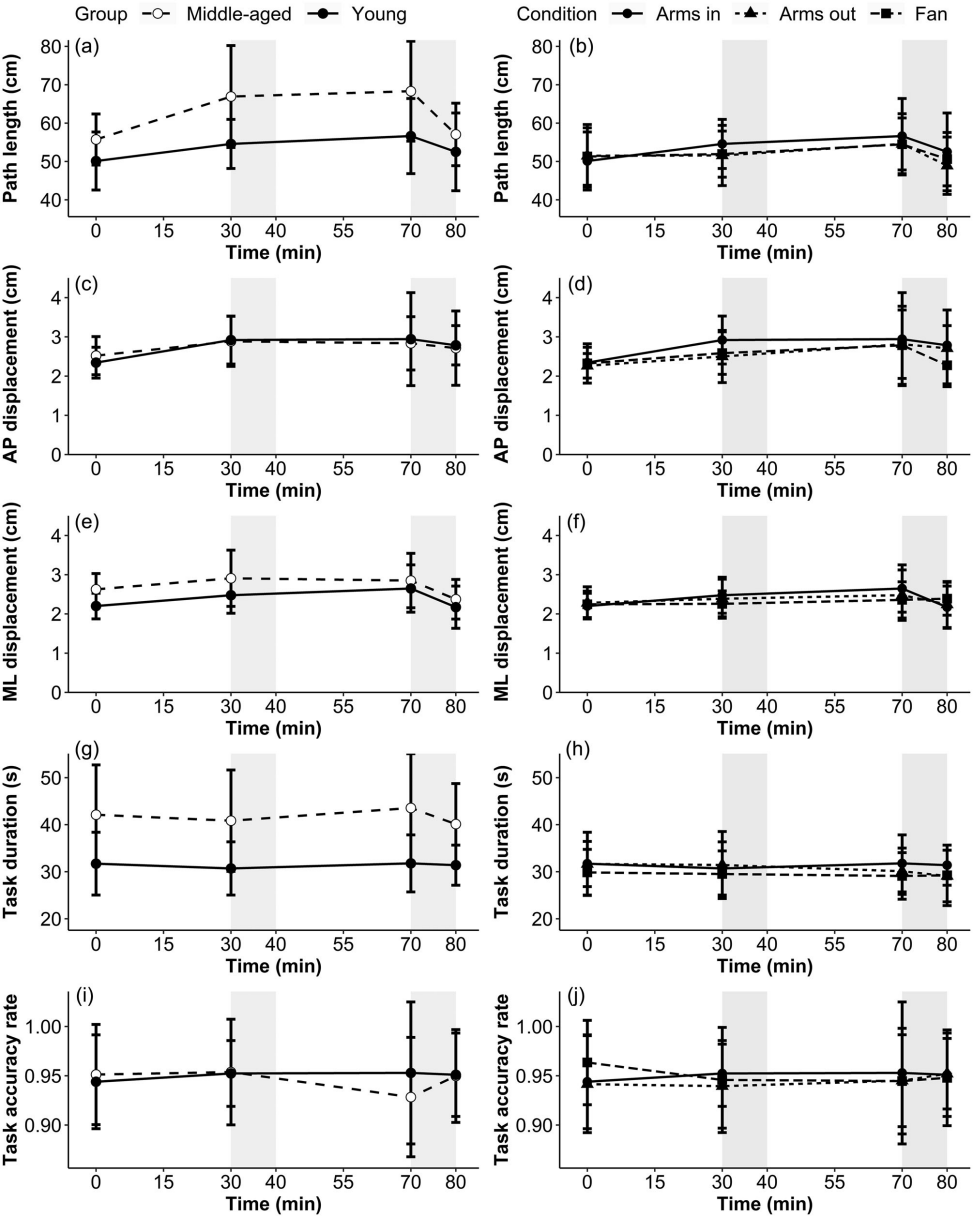
Postural sway and trail‐making test outcomes over time for the age comparison of path length (a), anteroposterior displacement (c), mediolateral displacement (e), task duration (g) and task accuracy rate (i); and condition comparison of path length (b), anteroposterior displacement (d), mediolateral displacement (f), task duration (h) and task accuracy rate (j) (*n* = 13 young, *n* = 17 middle‐aged). Shaded grey areas represent the non‐immersion periods during the protocol. Filled circles display the same data from younger participants in the Arms in condition for both the age (left‐hand panels) and condition (right‐hand panels). Abbreviations: AP, anteroposterior; ML, mediolateral.

All outcomes of postural sway displayed an increase after immersion, resulting in a main effect of time for group comparison (all *P* < 0.05), with middle‐aged participants having greater values than younger participants for path length (*P* = 0.01, *d* = 0.90; Figure 5a) and mediolateral displacement (*P* = 0.04, *d* = 0.59; Figure 5e), but no statistical effects being observed for group differences in anteroposterior displacement (*P* = 0.97, *d* = 0.03; Figure 5c) and no interaction effects (all *P* > 0.05, *R* ^2^_c_ < 0.20). In the condition comparison, for the postural sway outcomes, there was a significant main effect of time (*P* < 0.05) but not condition (*P* > 0.05) for path length (Arms out *d* = 0.21, Fan *d* = 0.15; Figure 5b) and anteroposterior displacement (Arms out *d* = 0.21, Fan *d* = 0.32; Figure 5d), with no observed effects for mediolateral displacement (*P* > 0.05, Arms out *d* = 0.03, Fan *d* = 0.11; Figure 5f).

### 3.5 Trail‐making test

For the trail‐making test, middle‐aged participants had a greater task duration than younger participants (Figure 5g), with similar error rates between groups resulting in a significant effect of group (*P* < 0.001, *d* = 1.16), but not time (*P* = 0.58) for task duration and no significant effects being observed for error rates for the age comparison (all *P* > 0.05, *d* = 0.07; Figure 5i). Likewise, for the condition comparison, no significant effects of time or condition (all *P* > 0.05; duration: Arms out *d* = 0.35, Fan *d* = 0.20; error rates: Arms out *d* = 0.11, Fan *d* = 0.02) were observed for the outcomes of the trail‐making test (Figure 5h,j).

### 3.6 Symptoms

During the immersion period, no participants reported severe heat illness symptoms of any type in any condition. In the younger group, three (3 of 13; 23%) participants reported moderate symptoms with four (4 of 13; 31%) participants experiencing no symptoms in any condition, and only two (2 of 17; 12%) middle‐aged participants reported moderate symptoms, with seven (7 of 17; 41%) experiencing no adverse symptoms. A summary of symptom frequencies can be seen in Table 1.

### 3.7 Exploratory correlations

Standing dizziness at 30 min in the Arms in condition showed weak‐to‐moderate negative correlations with standing SBP (*r* = −0.51, *P* = 0.004), age (*r* = −0.45, *P* = 0.01), affect (*r* = −0.43, *P* = 0.02) and seated SBP (*r* = −0.39, *P* = 0.04). However, when the data were analysed within each respective age group, no associations with standing dizziness were observed for any variable within the middle‐aged group, whereas in the younger group strong negative correlations with standing dizziness were observed for affect (*r* = −0.73, *P* = 0.005) and the change in seated SBP from rest (*r* = −0.70, *P* = 0.01), but no other variable. Sit‐Stand SBP displayed no significant correlations across the whole sample or within the middle‐aged group; however, strong correlations were observed within the younger group for heart rate (*r* = −0.84, *P* < 0.001) and thermal comfort (*r* = 0.72, *P* = 0.01). No significant associations were observed for Sit‐Stand DBP. Thermal comfort and affect at the 30 min time point were strongly associated across the whole sample (*r* = 0.79, *P* < 0.001) and within the younger (*r* = 0.79, *P* = 0.001) and middle‐aged (*r* = 0.67, *P* = 0.003) groups. Changes in seated DBP from rest were associated with the change in skin forehead temperature from rest (*r* = −0.39, *P* = 0.04), age (*r* = −0.45, *P* = 0.01), thermal comfort (*r* = −0.42, *P* = 0.02) and the change in seated SBP from rest (*r* = 0.58, *P* < 0.001) across the whole population, with none of these associations being significant within each age group. Likewise, changes in seated SBP were also associated with age (*r* = −0.54, *P* < 0.001) across the whole sample, but not within each age group.

## 4 DISCUSSION

This is the first study to consist of a comprehensive assessment of acute adverse responses to hot water immersion and the potential benefit of mitigation strategies. The specific purpose of this study was to compare the effects of whole‐body hot water immersion on orthostatic intolerance and selected physiological, perceptual, postural and cognitive responses in young and middle‐aged adults. Additionally, practical mitigation strategies were adopted to investigate whether they could reduce the adverse responses within the younger adults. Following Arms in hot water immersion, 94% of middle‐aged adults and 77% of younger adults reached the clinical threshold for orthostatic hypotension upon standing. This subsequently resulted in impaired postural control but had no impact on cognitive function for both age groups. Despite similar thermophysiological responses (Figure 3), middle‐aged adults tolerated the immersion and associated orthostasis well (Figure 4), whereas 30% of the younger cohort had to stop the session early owing to severe dizziness (15%) or heat intolerance (15%). Importantly, we found that these adverse responses could be largely negated with practical mitigation strategies, such as facial fan cooling or having the arms out of the water. Taken together, this study provides information about the relative risks, safety and potential mitigation strategies when using prolonged hot water immersion in young and middle‐aged adults.

Our study demonstrates that 30 min of immersion in 39°C water induces transient orthostatic hypotension, which was not present after 10 min of seated rest in ambient temperature (∼20°C; Figure 3). Postural hypotension was not asymptomatic as has been reported in other more modest passive heating studies (Lucas et al., 2008), in which internal temperature was raised by ∼0.5°C compared with 1.1°C in the present study. It is highly plausible that larger increases in rectal temperature result in an increased propensity and severity of negative symptoms. Furthermore, our data show that younger adults were more likely to present severe dizziness and thermal intolerance than middle‐aged adults. Heat‐induced orthostasis occurs owing to peripheral vasodilatation and blood pooling in the extremities, which can result in a reduction in cerebral perfusion (Schlader et al., 2016). Measurements of cerebral perfusion might have provided some additional mechanistic insight into the findings of our study, but it should be acknowledged that heat‐induced reductions in cerebral perfusion do not predict subsequent reductions in orthostatic tolerance (Lee et al., 2013). Reductions in cerebral perfusion can also impact other central processes, and in this regard, we found that postural control, but not cognitive function, was impaired after passive heating in both age groups. To our knowledge, this is the first study to demonstrate impaired postural control after hot water immersion. In the majority of cases, the degree of heat‐induced orthostatic hypotension observed in the present study was a harmless event, yet the manifestation of dizziness and impaired postural control is an important finding because this might lead to a transient window of increased risk of falls (Johansson et al., 2017). Indeed, analysis of autopsies after bath‐related deaths has identified orthostatic hypotension upon standing as the most likely cause of falls and mortality (Oshima et al., 2020). Based on such findings, we urge caution before longer bathing durations and higher temperatures of water immersion when unsupervised without effective mitigations.

Despite comparable changes in thermophysiological measures, middle‐aged adults perceived thermal sensation to be lower, as well as having higher levels of thermal comfort and basic affect (Figure 4). The absence of thermophysiological differences between age groups was somewhat unexpected given that biological ageing can impair the ability of the body to dissipate heat, for example, by cutaneous vasodilatation and sweat production (Kenney & Munce, 2003; Stapleton et al., 2015). It is conceivable that the largely uncompensable environment might have lessened the capacity of such mechanisms to support thermoregulation in the young adults. However, the observed difference in perceptual responses is consistent with previous research showing a decrease in peripheral sensitivity to the heat with age (Inoue et al., 2016; Stevens & Choo, 1998). The higher thermal comfort scores in the middle‐aged adults might explain why no participants in that group terminated the protocol owing to thermal intolerance. This suggests that hot water immersion might be a more appealing intervention for middle‐aged adults and that mitigation strategies are not always necessary. However, future studies might investigate the behavioural component of thermoregulation in the context of hot water immersion, because the lack of peripheral perceptual awareness might cause middle‐aged adults to be less likely to carry out thermoregulatory behaviours, such as session termination, that might result in worse outcomes for heat‐related adverse events.

In the younger adults, the present study showed the use of facial fan cooling and non‐submersion of the arms to be effective mitigation strategies in reducing the prevalence of orthostatic hypotension and severe dizziness, in addition to improving thermal tolerance to the protocol. This improved tolerance was accompanied by reduced thermophysiological strain and improved levels of thermal comfort and basic affect. Likewise, previous research has demonstrated improved perceptual responses to 42°C water immersion with upper body fan cooling, with the authors suggesting that this might lead to improved adherence to repeated exposures without impacting the beneficial adaptive effects of the acute interleukin‐6 response (Mansfield et al., 2021). However, vascular adaptation following passive heating is dependent on episodic increases in shear stress (Carter, Spence, Atkinson, Pugh, Naylor et al., 2014), with these acute vascular responses to heating being determined, in part, by skin and core temperatures (Carter, Spence, Atkinson, Pugh, Cable et al., 2014; Coombs et al., 2021). Therefore, some beneficial effects of hot water immersion might be reduced by the use of mitigation strategies designed to improve tolerability and adherence. Indeed, both thermal intolerance (Zurawlew et al., 2016) and orthostatic intolerance (Shvartz et al., 1975) are improved after heat acclimation. This might mean that tolerability and the acute adaptive stimulus should be considered together when deciding on the most appropriate protocol for hot water immersion. Accordingly, future research should investigate the effects of these mitigation strategies on acute responses thought to be beneficial to adaptation and whether removal of the mitigation strategies to allow for progression in the hot water immersion stimulus as tolerance improves with repeated exposures. Future studies might also consider strategies to reduce the degree of postural hypotension, therefore reducing risk, which do not reduce other potentially beneficial physiological responses.

The ability to predict which individuals are at greater risk of severe dizziness or syncope before standing would enable additional precautionary measures to be implemented to ensure safe exit from the water. Traditionally monitored variables, such as rectal temperature or heart rate, showed no relationship with standing dizziness in the present study. Instead, the best correlate of standing dizziness across all participants in the Arms in condition at 30 min was standing SBP, which showed a moderate negative relationship with standing dizziness (*r* = −0.51). In our study, we found that age accounted for 20% and standing SBP for 25% of the variance in standing dizziness. It is possible that the lower absolute SBP in the young group might explain their increased susceptibility to developing symptoms of dizziness. A further explanation for different levels of symptom presentation between younger and middle‐aged adults in our study might be attributable to the higher levels of muscle sympathetic nerve activity typically seen in older adults (Matsukawa et al., 1998). Cui et al. (2011) have reported that individuals with a high muscle sympathetic nerve activity during orthostasis under heat stress also had the highest orthostatic tolerance. Indeed, these two explanations are likely to be linked, because age‐related increases in blood pressure are likely to be driven by higher muscle sympathetic nerve activity.

In the younger adults, who were far more prone to severe dizziness, basic affect and the change in seated SBP from baseline accounted for 53 and 49%, respectively, of the variance in the degree of dizziness experienced upon standing. No variables were related to standing dizziness in the middle‐aged cohort, which might be attributable to the small range of responses in standing dizziness observed (9 of 17; 53% reported a dizziness score of 0 at 30 min). Therefore, in the absence of more detailed measures, basic affect and the change in seated SBP from baseline might be useful precautionary measures for judging the risk of severe dizziness in young adults; however, further research is required to confirm this relationship and whether it can be expanded to different populations.

It should be acknowledged that this study is not without its limitations. Owing to the use of whole‐body hot water immersion, we were unable to measure blood pressure continuously; therefore, it is possible that we have slightly underestimated the decrease in blood pressure upon standing. Nonetheless, our data do show large hypotensive responses in most of the participants. Nevertheless, we were probably underpowered to detect some interaction effects and hope that our data can be used to power future studies appropriately. Although our findings cannot be applied directly to other doses of hot water immersion, different modes of passive heating or other populations, they highlight an important safety issue that we feel has been somewhat overlooked to date. Indeed, the described prevalence and severity of adverse responses in the present study might have been greater without multiple elements of the present experimental protocol, such as the 10 min in ambient conditions after 30 min of hot water immersion, the bolus of water drunk at the start and the instructions to stand up gradually after immersion, offering some mitigation to potential adverse reactions. Moreover, given that we included only relatively healthy (low‐risk) individuals owing to the possibility of adverse events, our results cannot be extrapolated to clinical populations, who might have divergent responses. Future studies should investigate the relative risk and tolerability of hot water immersion with other groups who have the most to gain from the potential health benefits, such as those with type 2 diabetes, spinal cord injury and elderly populations, all of whom are known to have impaired thermoregulatory responses (Kenney & Munce, 2003; Schlader et al., 2016).

## 5 CONCLUSIONS

In summary, this study showed that 30 min of whole‐body hot water immersion at 39°C induced transient orthostatic intolerance. This resulted in impaired postural control but had no impact on cognitive function for both young and middle‐aged adults. Hot water immersion was well tolerated by middle‐aged adults, but younger adults reported a greater frequency and severity of dizziness and heat intolerance. In certain individuals, this could result in an increased risk of adverse events, such as falling or syncope when standing or exiting the water, highlighting the need for caution before such protocols are performed in an unsupervised environment. Finally, we demonstrated that these adverse responses in young adults could be mitigated by practical mitigation strategies, such as using a fan or not immersing the arms.

## AUTHOR CONTRIBUTIONS

Tom Cullen, Campbell Menzies, Charles Steward, Christopher Pugh, Douglas Thake, Neil Clarke and Mathew Hill were responsible for the conception and design of the study. Campbell Menzies, Charles Steward, Tom Cullen and Amy Harwood were responsible for data acquisition, while Doug Thake, Neil Clarke, Christopher Pugh and Mathew Hill also assisted in interpretation of the data. All authors contributed to drafting or revision of the written work, approved the final version and agree to be accountable for all aspects of the work in ensuring that questions related to the accuracy or integrity of any part of the work are appropriately investigated and resolved. All persons designated as authors qualify for authorship, and all those who qualify for authorship are listed.

## CONFLICT OF INTEREST

None declared.

## FUNDING INFORMATION

The authors received no external funding to support the work.

## Supporting information



Statistical Summary Document

DataSet1

DataSet2

## Data Availability

All raw data and the code used to analyse it is provided as supplementary information.
